# T Cell Motility─How Is It Regulated?

**DOI:** 10.3389/fimmu.2020.588642

**Published:** 2020-12-11

**Authors:** Karl-Gösta Sundqvist

**Affiliations:** Department of Laboratory Medicine, Division of Clinical Immunology, Karolinska Institute and Clinical Immunology and Transfusion Medicine Karolinska University Hospital, Stockholm, Sweden

**Keywords:** lipoprotein receptor-related protein 1, thrombospondin-1, chemokine receptors, integrins, adhesion, motility

## Introduction

Cell motility and its regulation attract enormous interest. This is particularly evident in immunology with a literature explosion on T cell motility in search for antigen, surveillance of the organism for infected cells and cancer cells but also pathological infiltration of T cells in inflammatory diseases. The prevailing concept is that cell motility, including ameboid-like motility in T cells, is driven by integrin and chemokine receptor signaling and reorganization of actin (1–7). There is, however, no consensus on the mechanism of ameboid movement in general, on the precise mechanisms of cell motion, and how T cells adapt their motility to different environments and activities (6–13). It also remains unclear how cell polarity, the basis of directed cell movements, is directed (14), and why T cell motility and extravasation, as well as motility of amoebae, are dependent on protein synthesis (15–18).

Contemporary motility research is focusing on outside-in signaling through chemokine receptor and integrin transmembrane signaling to the cytomusculature (1–14) or on a regulatory role of cytomuscular components on plasma membrane molecules (19). However, motility of a search and surveillance cell, like the T cell, is likely to depend on cell-intrinsic regulation and to be adaptive to its environment. Here I discuss cell-intrinsic regulation of T cell polarity, motility and adhesion through an intra-plasma membrane crosslinking cascade, which promotes integrin and chemokine receptor effects on motility. This cascade is triggered by the transmembrane receptor low density lipoprotein receptor-related protein 1 (LRP1) and co-receptor calreticulin through their ligand thrombospondin-1 (TSP-1). This cell-intrinsic mechanism senses and adapts motility to the microenvironment and antagonizes integrin-dependent contacts through shedding of LRP1. Antigen stimulation targets LRP1 and TSP-1 suggesting that the cell-intrinsic motility mechanism has a central functional role in T cells.

LRP1 and TSP-1 are uniquely equipped for crosslinking interactions due to their size and numerous binding sites for other molecules (20, 21). LRP1 consists of an α-chain (515 kDa) containing ligand-binding domains, a β-chain (85 kDa) containing the transmembrane domain and the cytoplasmic tail. TSP1 is a 450-kDa adhesive glycoprotein composed of three identical disulfide-linked polypeptide chains that display binding sites for various cell surface receptors and other molecules including LRP1, calreticulin, integrins, CD47, heparan sulfate proteoglycans and fibronectin.

## Cell Surface Crosslinking–A Fundamental Motogenic Principle

The formation of motile active cell edges requires actin nucleation and polymerization by preformed actin filaments and nucleators, including the Arp2/3 complex, WASP and formins and linkage to the plasma membrane through ezrin-moesin-radixin. Cell surface receptors depend on this association to the actomyosin system for their expression, localization and function. Actomyosin contractility has a central role for cell motility and is generated by myosin-exerted force on actin filaments.

A key question is how the actomyosin machinery in cells with ameboid-like motility and with a key role to search and surveil is regulated. In ameboid three-dimensional migration myosin is thought to start the motile process. However, there is substantial evidence that actin rearrangements and motility are directed at the level of the plasma membrane through crosslinking of cell surface receptors (22–25). Cytoplasmic spreading on insolubilized ligands depends on the crosslinking principle. The power of this mechanism is evident by its independence of the specificity of the ligands (26) although physiological crosslinking in adherent cells is mediated through integrins. Ameboid-like T cell motility in three-dimensional environments independent of adhesive contacts may therefore be postulated to require cell-intrinsic crosslinking of plasma membrane receptors.

## Motility and Sensing Through a Crosslinking Cascade

In support of the concept that motility is directed at the plasma membrane level, early work showed that T cell motility is associated with surface expression of a complex of endogenous high-molecular weight proteins and a 55 kDa protein maintained by contact with type 1 collagen matrices and other cells but lost by contact with plastic (16, 27). Subsequently, the endogenous high molecular weight proteins LRP1 and TSP-1 have been shown to regulate T cell motility and integrin-dependent adhesion through collaboration with other proteins, including the 50 kDa protein calreticulin, CD47 and CD26 (28–34). Zinc chloride stabilizes this complex of motogenic cell surface molecules while enhancing motility (28).

**Figure 1:**
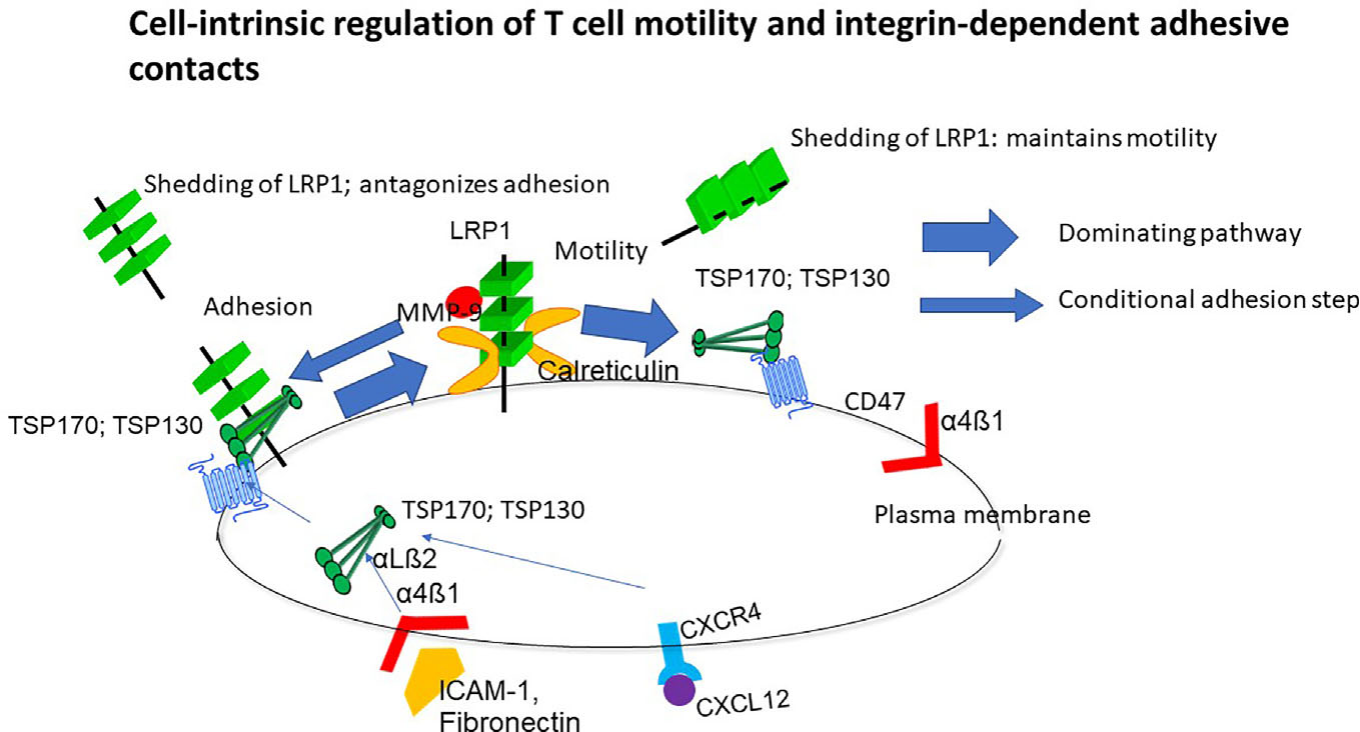
Overview of the cell-intrinsic motility mechanism. This mechanism is dependent on non-adhesive environmental support but upregulated in response to ligation of integrins and chemokine receptors while preventing persistent adhesion. Lipoprotein receptor-related protein 1 (LRP1)-calreticulin complexes trigger motility through the NH-terminal domain of thrombospondin-1 (TSP-1) and a coupled interaction of the COOH-terminal with CD47. T cells are hence programmed to move, and this behavior is prioritized before persistent adhesion unless LRP1 shedding is abrogated (34). TSP-1 reduces shedding of LRP1 and this reduction is associated with stimulation of motility.

LRP1 and associated calreticulin trigger a polarized motile morphology and migration through the NH-terminal region of TSP-1 and interaction of its distal COOH-terminal region with CD47, but TSP-1 may also crosslink other cell surface receptors (**Figure 1**). On the contrary, TSP-1 does not trigger motility through LRP1-calreticulin indicating that the motogenic crosslinking cascade is directed and implicates a regular controlled process. This directed crosslinking may explain how T cell polarity is controlled. LRP1 induces motility by binding intact 170 kDa TSP1 and a 130 kDa TSP-1 fragment and maintains the localization of TSP-1 within the plasma membrane of T cells (30, 32). Inhibition of LRP1 hence changes the distribution of TSP-1 from discontinuous patchy on cells with different motile morphologies to a cap-like localization on spherical cells. This association of LRP1 and TSP-1 probably explains why silencing, or blocking ligand binding of LRP1, inhibit T cell motility (28–34). In contrast to endogenous TSP-1, exogenous TSP-1 or a peptide mimetic of its NH-terminal calreticulin binding site inhibit motility (28), which strongly supports that motility is triggered by endogenous LRP1-calreticulin complexes in *cis via* TSP-1. It is also worthy to note that MMP-9 stimulates three-dimensional T cell motility in an extracellular matrix model independent of proteolysis (35), which may reflect that it is a ligand for LRP1 (36) and hence may stimulate the cell-intrinsic motogenic cascade.

T cell motility depends on the LRP1-regulated motility mechanism (28–34) and is markedly higher in cells contacting collagen matrices and other cells than plastic (16, 27), which implies that this mechanism senses the cellular microenvironment. This conclusion is reinforced by the fact that the LRP1-regulated motility mechanism maintains a T cell phenotype for scanning of surrounding structures by preventing cytoplasmic spreading with disappearance of microvilli and arrest in cells contacting integrin ligands (28, 31, 34). The LRP1-regulated motility mechanism further integrates sensing of the T cell microenvironment through integrin and chemokine receptors (30, 32). This may explain how cells coordinate environmental signaling through multiple, often redundant receptors.

## A Labile Mechanism for Sensing and Motility Through Dependence of Protein Synthesis on Environmental Support

T cell motility and extravasation, as well as motility of ameba, depend on protein synthesis although the driving of motility by actin reorganization and signaling does not (15–18). A clue to the dependence of motility on protein synthesis is that cell surface proteins sensing the environment and regulating motility have a high turnover (16, 28, 29). Shedding of LRP1 (34) may contribute to this but also internalization and degradation. It is reasonable to assume that contact with collagen matrices and other cells maintains motility by supporting synthesis of motogenic proteins since protein synthesis requires environmental support (37). The dependence on sensing of the environment further suggests that the LRP1-directed motility mechanism is responsible for the dependence of motility on confinement.

## Three-Dimensional (3D) T Cell Migration Depends on the LRP1- and TSP-1 Directed Motility Mechanism

It is important to define the influence on T cell motility of the LRP1-directed motility mechanism and signaling through integrins and chemokine receptors. This issue is well illustrated by experiments studying T cells in a 3D environment. T cell motility into a collagen matrix hence depends on the LRP1-directed motility mechanism (28–34). In contrast, signaling through integrin and chemokine receptors *does* not give T cells ability to enter 3D matrices but enhances existing capacity to enter such matrices (38). However, in T cells with capacity to enter collagen matrices, infiltration is markedly enhanced through expression of collagen-binding integrins (39). T cells expressing collagen receptors rapidly penetrate and unlike fibroblasts and macrophages do not undergo cytoplasmic spreading on top of the collagen. This is probably important for T cell surveillance and protection against infections in non-lymphoid organs. Collagen-binding integrins are hence crucial for T cell infiltration in tissues affected by inflammatory diseases (40, 41). It should be mentioned that T cell motility in collagen matrices has been reported to be integrin-independent (42). However, this conclusion may reflect use of cells lacking collagen-binding integrins (ConA-stimulation for 3 days) since expression of these integrins require long-term activation and lectins have been reported to inhibit integrins (43).

## Adhesion Control

The LRP1-regulated motility mechanism promotes integrin-dependent adhesive contacts (29). However, shedding of LRP1 maintains T cell motility during contact with integrin ligands (34). Accordingly, abrogation of shedding by a broad-spectrum metalloprotease inhibitor (34) causes accumulation of LRP1 and TSP-1 at the cell edge, firm integrin-dependent adhesion, apolar cytoplasmic spreading, and motility arrest. In contrast, the intrinsic capacity of the LRP1-regulated motility mechanism to reduce shedding is *set* to merely counterbalance adhesion and prioritize motility.

The chemokines CXCL12 and CCL5, and contact with integrin ligands, stimulate transport of TSP‐1 and LRP1 to the cell surface (30, 32). CXCL12 and integrin ligands induce surface expression of intact TSP‐1 and a 130 000 MW TSP‐1 fragment, which associate with LRP1, while preventing expression of 110,000 MW TSP‐1 fragment. This fragment does not associate with LRP1 and correlates with de-adhesion. This suggests that differential cell surface expression of TSP-1 and TSP-1 fragments regulate T cell motility and adhesive contacts in collaboration with LRP1.

## A Mechanism for Search and Surveillance

The LRP1-regulated motility mechanism hence combines an ability to sense environmental structures independent of conventional receptors and adapt to this sensing. Sensing and motility are hence tightly coupled indicating that this mechanism is important for T cell search and surveillance. Shedding of LRP1 may further be critical to promote search for antigen by preventing persistent adhesive contacts and may prevent this large molecule from interference with TCR-pMHC interactions. The ability to integrate and respond to integrin and chemokine receptor signals further endows this mechanism with a global sensing capacity consistent with a key role for T cell search and surveillance. Integrin and chemokine receptor signals stimulate this mechanism through enhanced TSP-1 transport to the cell surface antagonizing shedding of LRP1 (30, 32, 34). This implies that a labile mechanism for sensing and motility is enhanced by integrin and chemokine receptor signals.

## Ligation of TCR/CD3 and CD28 Targets LRP1 and TSP-1

TCR-induced arrest upon antigen recognition, cytoplasmic spreading and actin polymerization are considered central in T cell biology and thought to represent decision to enter an activation program (44). Collaboration between TCR/CD3 ligation and ligation of LFA-1 enhances transport of TSP-1 to the cell surface, which antagonizes shedding of LRP (29, 30, 34). Ligation of CD28 also inhibits shedding of LRP1 but through a distinct mechanism (35). Antigen stimulation promotes contact between T cells and antigen-presenting cells probably through upregulated TSP-1 and reduced LRP1 expression (34). This indicates that the cell-intrinsic regulation of motility and integrin-dependent adhesion is important for TCR-induced activation.

## Therapeutic Implications

The cell-intrinsic motility mechanism represents a therapeutic target for interference with T cell activity, for example in inflammatory diseases. It is interesting in this context that the cornerstone therapy for inflammatory diseases, methotrexate, stimulates this motility mechanism. This is consistent with an immunoregulatory function of this mechanism and suggests that methotrexate, at the relatively low concentrations used to treat inflammatory diseases, is immunoregulatory rather than inhibitory (45).

## Author Contributions

The author confirms being the sole contributor of this work and has approved it for publication.

## Funding

This work was supported by the Swedish Cancer Foundation and the Swedish Research Council.

## Conflict of Interest

The author declares that the research was conducted in the absence of any commercial or financial relationships that could be construed as a potential conflict of interest.
